# Antibacterial Activity of the Non-Cytotoxic Peptide (p-BthTX-I)_2_ and Its Serum Degradation Product against Multidrug-Resistant Bacteria

**DOI:** 10.3390/molecules22111898

**Published:** 2017-11-04

**Authors:** Norival A. Santos-Filho, Rafaela S. Fernandes, Bruna F. Sgardioli, Matheus A. S. Ramos, Julia P. Piccoli, Ilana L. B. C. Camargo, Tais M. Bauab, Eduardo M. Cilli

**Affiliations:** 1 Instituto de Química, Universidade Estadual Paulista (UNESP), Araraquara-SP 14800-060, Brazil; juliappiccoli@gmail.com; 2 Instituto de Física de São Carlos, USP—Universidade de São Paulo, São Carlos-SP 13563-120, Brazil; rafaela.fernandes@usp.br (R.S.F.); sgardiolibruna@yahoo.com.br (B.F.S.); ilanacamargo@ifsc.usp.br (I.L.B.C.C.); 3 Faculdade de Ciências Farmacêuticas, Universidade Estadual Paulista (UNESP), Araraquara-SP 14800-903, Brazil; matheusramos_91@hotmail.com (M.A.S.R.); tmbauab@gmail.com (T.M.B.)

**Keywords:** (p-BthTX-I)_2_, multidrug-resistant bacteria, biofilm, antimicrobial peptides

## Abstract

Antimicrobial peptides can be used systemically, however, their susceptibility to proteases is a major obstacle in peptide-based therapeutic development. In the present study, the serum stability of p-BthTX-I (KKYRYHLKPFCKK) and (p-BthTX-I)_2_, a p-BthTX-I disulfide-linked dimer, were analyzed by mass spectrometry and analytical high-performance liquid chromatography (HPLC). Antimicrobial activities were assessed by determining their minimum inhibitory concentrations (MIC) using cation-adjusted Mueller–Hinton broth. Furthermore, biofilm eradication and time-kill kinetics were performed. Our results showed that p-BthTX-I and (p-BthTX-I)_2_ were completely degraded after 25 min. Mass spectrometry showed that the primary degradation product was a peptide that had lost four lysine residues on its C-terminus region (des-Lys^12^/Lys^13^-(p-BthTX-I)_2_), which was stable after 24 h of incubation. The antibacterial activities of the peptides p-BthTX-I, (p-BthTX-I)_2_, and des-Lys^12^/Lys^13^-(p-BthTX-I)_2_ were evaluated against a variety of bacteria, including multidrug-resistant strains. Des-Lys^12^/Lys^13^-(p-BthTX-I)_2_ and (p-BthTX-I)_2_ degraded *Staphylococcus epidermidis* biofilms. Additionally, both the peptides exhibited bactericidal activities against planktonic *S. epidermidis* in time-kill assays. The emergence of bacterial resistance to a variety of antibiotics used in clinics is the ultimate challenge for microbial infection control. Therefore, our results demonstrated that both peptides analyzed and the product of proteolysis obtained from (p-BthTX-I)_2_ are promising prototypes as novel drugs to treat multidrug-resistant bacterial infections.

## 1. Introduction

Infectious diseases are one of the main causes of death worldwide, particularly in developing countries. The increase in bacterial resistance to conventional antibiotics is a consequence of their widespread and uncontrolled use. Thus, the search for new therapeutic molecules to control microbial infections is highly important for tackling this challenge [1,2,3,4].

Antimicrobial peptides (AMPs) are a potential alternative treatment to traditional antibiotics owing to their efficacy, safety, enormous diversity, and broad-spectrum activity against a wide range of microorganisms, including oral bacteria [5,6], multidrug-resistant bacteria [7], fungi [8], and viruses [3].

An increasing number of antimicrobial peptides have been isolated and studied from different natural sources [9,10]. AMPs are usually classified into four groups according to their structural characteristics: (1) cysteine-rich and β-sheet AMPs (α- and β-defensins); (2) AMPs possessing α-helices (LL-37 cathelicidin, cecropins, and magainins); (3) AMPs with extended structures (rich in glycine, proline, tryptophan, arginine, and/or histidine); (4) and AMPs containing peptide “loops” formed by single disulfide bonds (bactenecin) [11].

AMPs are among the several compounds purified and characterized from animal venoms, e.g., a potent AMP isolated from the venom of the spider *Lachesana tarabaevi* [12] and a 45-residue, α-helix cationic, and cysteine-rich peptide from the venom of the scorpion *Parabuthus schlechteri* [13]. Another AMP isolated from *Bothrops jararaca* venom with the molecular weight of 1.37 kDa inhibits the growth of several fungi and bacteria through a membrane-destabilizing mechanism [14].

Based on the antimicrobial activity described for *B. asper* Lys49 myotoxin [15], novel peptides derived from its C-terminal region have been studied. The C-terminal peptide, 115–129, of myotoxin II demonstrated bactericidal activity similar to that of the parent protein through a mechanism involving membrane permeabilization [15]. Another study demonstrated that synthetic cationic peptides derived from the C-terminal region (residues 115–129) of *B. brazili* myotoxin II were microbicidal against *Escherichia coli*, *Candida albicans*, and *Leishmania* spp. [16].

Bothropstoxin-I (BthTX-I) is a single chain protein of 13.7 kDa, isoelectric point (pI) 8.2, 121 amino acid residues, and seven disulfide bonds isolated from the venom of the snake *B. jararacussu* [17]. In a previous study, this basic myotoxin showed microbicidal action by increasing the membrane permeabilization of microorganisms [18]. Based on the antimicrobial activity of BThTX-I and on the premise that the C-terminal peptide of Lys49 myotoxin can reproduce the microbial activity of the parent protein, our research group synthesized and characterized a peptide derived from the C-terminal region of the Phospholipase A_2_ (PLA_2_) homologue bothropstoxin-I (BthTX-I) (p-BthTX-I, sequence: KKYRYHLKPFCKK) and its disulfide-linked dimer (p-BthTX-I)_2_ [19]. In this study, p-BthTX-I and (p-BthTX-I)_2_ showed antimicrobial activity against both gram-negative and gram-positive bacteria, with the peptide’s dimeric form and the Cys residue essential for its activity. Moreover, p-BthTX-I and (p-BthTX-I)_2_ did not promote lysis or form membrane pores, did not interact with membranes, and did not present activity against *C. albicans*, erythrocytes, epithelial cells, or macrophages, showing a possible specificity against prokaryotic cells, which highlighted the therapeutic potential of these molecules [19].

Herein, the serum stability of (p-BthTX-I)_2_ was analyzed, and the peptide was determined to form stable, promising degradation products (des-Lys^12^/Lys^13^-(p-BthTX-I)_2_) that retained the biological activity of the parent peptide. Additionally, we extended the analysis of the antibacterial activity of these peptides against a variety of bacteria, including multidrug-resistant strains. The peptides were evaluated for their abilities to degrade biofilms formed by *Staphylococcus epidermidis* ATCC35984, a strain known to produce biofilms, and their bactericidal activities against the planktonic form of the same strain by time-kill kinetics analyses.

## 2. Results

**Figure 1:**
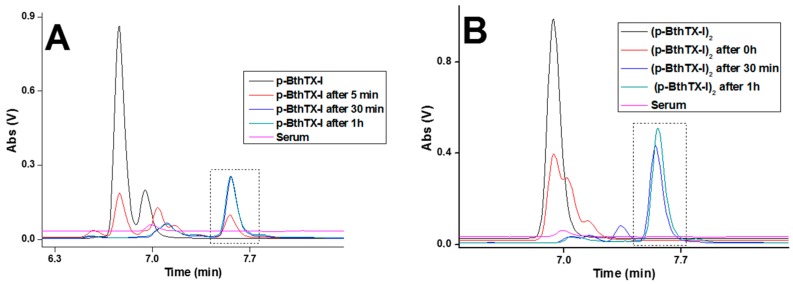
Serum stability assays by analytical high-performance liquid chromatography (HPLC). (**A**) p-BthTX-I and (**B**) (p-BthTX-I)_2_ were incubated with human serum for the indicated times. Analytical HPLC was performed on a Shimadzu (Kyoto, Japan) system using a C18 column (4.6 × 150 mm, Phenomenex) with a linear gradient of solvent B (5–95%) at 1 mL/min for 15 min (Solvent A: 0.045% trifluoroacetic acid (TFA) in water. Solvent B: 0.036% TFA in acetonitrile). Marked boxes in the figures show the same degradation product produced by both the peptides.

To determine the stabilities of the peptides, high-performance liquid chromatography (HPLC) analyses using a C18 reverse phase column were performed. The peptide p-BthTX-I and its disulfide linked dimer (p-BthTX-I)_2_ exhibited retention times (RT) of 6.7 and 6.9 min, respectively (Figure 1). After incubation with human serum, both peptides showed accelerated rates of initial degradation (*t* = 5 min), which were clearly demonstrated by the decreased integrated peak areas (Figure 1, red line). These data also indicated that complete degradation of the dimer occurred after 30 min (Figure 1, blue line). Post degradation (30 min and 1 h, respectively), both peptides exhibited peaks that eluted at 7.5 min (Figure 1, marked box), suggesting the same degradation product for both the monomeric and dimeric forms of the peptide. Additionally, we have shown in a previous work that dimerization is swiftly initiated when the monomeric p-BthTX-I peptide is incubated in the culture medium [19], suggesting that only the dimer (p-BthTX-I)_2_ likely occurs in human serum.

**Figure 2:**
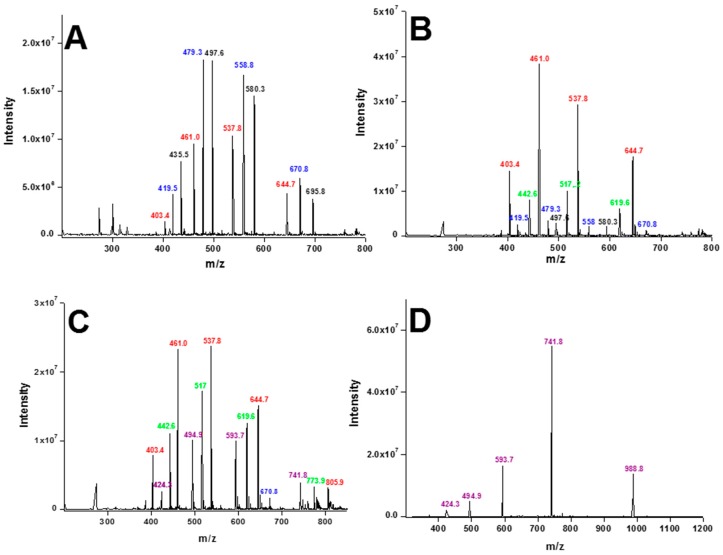
Serum stability analyses by mass spectrometry. Analyses of peptides and degradation products were performed after (**A**) 5 min, (**B**) 20 min, (**C**) 30 min, and (**D**) 120 min of incubation in human serum. Molecular ions: intact (p-BthTX-I)_2_, black; (p-BthTX-I)_2_ minus one Lys residue, blue; (p-BthTX-I)_2_ minus two Lys residues, red; (p-BthTX-I)_2_ minus three Lys residues, green; and (p-BthTX-I)_2_ without four Lys residues, purple.

To identify the serum degradation products of the peptides, mass spectrometry analyses were performed. After 5 min of incubation in serum, (p-BthTX-I)_2_ clearly produced two degradation products (Figure 2A). Two different peptides were identified: one that had lost a single lysine residue (Figure 2A, molecular mass segments in blue) and another that lost two lysine residues (Figure 2A, molecular mass segments in red). Numbers in black are relative of the intact dimeric peptide.

After 10 min, the peptide that had lost two lysine residues was the major peptide product; however, the parent peptide and the other degradation product were still detected. Within 20 min of incubation, a peptide that had lost three lysine residues was identified (Figure 2B, green numbers). After 30 min of incubation, a combination of peptide products having lost two, three, and four lysine residues could be observed (Figure 2C). At later times (120 min), only the peptide product without the four lysine residues remained (Figure 2D, purple numbers); this result was in agreement with the stable degradation product peak observed by HPLC. This peptide remained intact after 12 and 24 h; however, further degradation products were observed after 30 h of incubation (data not shown), highlighting the high stability of the degradation product without the four Lys residues.

To identify the serum degradation peptides from (p-BthTX-I)_2_, four analogues were synthesized and dimerized by interchain disulfide bond formation (Cys–Cys) following an air oxidation procedure. The following peptides were purified and characterized by analytical HPLC and electrospray mass spectrometry (Table 1): des-Lys^13^-(p-BthTX-I)_2_ [sequence: (KKYRYHLKPFCK)_2_], des-Lys^12^/Lys^13^-(p-BthTX-I)_2_ [sequence: (KKYRYHLKPFC)_2_], des-Lys^1^/Lys^2^-(p-BthTX-I)_2_ [sequence: (YRYHLKPFCKK)_2_], and des-Lys^1^-(p-BthTX-I)_2_ [sequence: (KYRYHLKPFCKK)_2_].

**Figure 3:**
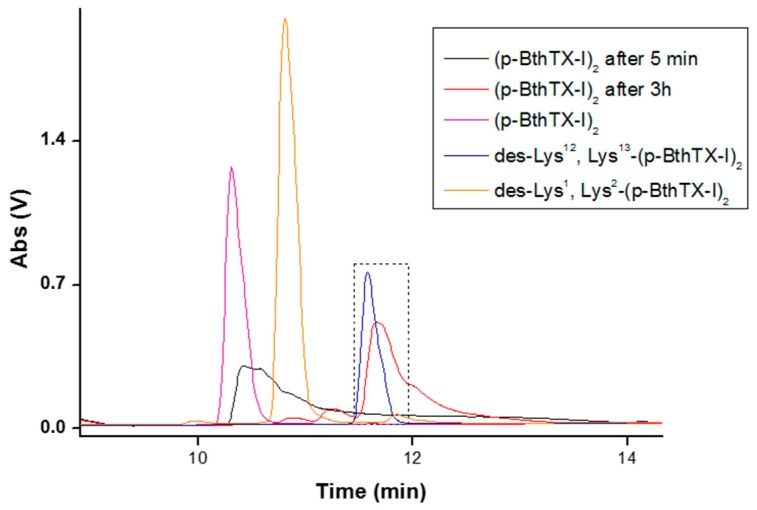
Analytical HPLC comparing the peaks of the peptides (p-BthTX-I)_2_, des-Lys^12^/Lys^13^-(p-BthTX-I)_2_, and des-Lys^1^/Lys^2^-(p-BthTX-I)_2_ to the RT of peaks of the stable serum degradation product of (p-BthTX-I)_2_. Samples were eluted at 5 min and 3 h. Analytical HPLC was performed on a Shimadzu system using a C18 column (4.6 × 150 mm, Phenomenex) with a linear gradient of solvent B (5–95%) at 1 mL/min for 30 min (Solvent A: 0.045% trifluoroacetic acid (TFA) in water. Solvent B: 0.036% TFA in acetonitrile). Marked box indicates the peak of des-Lys^12^/Lys^13^-(p-BthTX-I)_2_ and the stable degradation peptide produced.

To confirm the identity of the stable product from (p-BthTX-I)_2_, serum degradation assays were performed again and analyzed by HPLC using a C18 reverse phase column. The analytical HPLC profiles of the degradation products was compared with the retention time (RT) of the synthesized peptides (des-Lys^13^-(p-BthTX-I)_2_, des-Lys^12^/Lys^13^-(p-BthTX-I)_2_, des-Lys^1^/Lys^2^-(p-BthTX-I)_2_, and des-Lys^1^-(p-BthTX-I)_2_). In agreement with mass spectrometry results, the stable product was confirmed to be the peptide without four lysine residues on its C-terminus (des-Lys^12^/ Lys^13^-(p-BthTX-I)_2_) (Figure 3).

Because the peptides p-BthTX-I and (p-BthTX-I)_2_ exhibited specific antibacterial activities, we next tested the antibacterial and antifungal activities of the synthesized peptides against *E. coli*, *Staphylococcus aureus*, and *C. albicans* (Table 2) as well as a panel of multidrug-resistant clinical strains (Table 3). Of the total 20 bacterial strains tested, p-BthTX-I presented an antibacterial activity against *S. epidermidis* ATCC35984, *S. aureus* strains SA16, SA33, SA88 and SA90, *Enterococcus faecium* strains VRE16 and HSJRP8, *Klebsiella pneumoniae* NDM-1 and *E. coli* ATCC35218. The dimeric peptide was active against the same strains as its monomeric form and additionally showed an action against *S. aureus* ATCC25923, multidrug-resistant *K. pneumoniae* strains ATCC700603 and ATCC BAA1705 also against *E. coli* strains ATCC25922 and CA4, a commensal bacterium (Table 3). On the other hand, the serum degradation product, des-Lys^12^/Lys^13^-(p-BthTX-I)_2_, exhibited superior antimicrobial effect against *S. aureus* strains ATCC25923, SA16 and SA33, *E. faecium* strains VRE16 and HSJRP8, *K. pneumoniae* ATCC BAA1705 and *E. coli* strains ATCC35218 and CA4 (Table 3).

**Figure 4:**
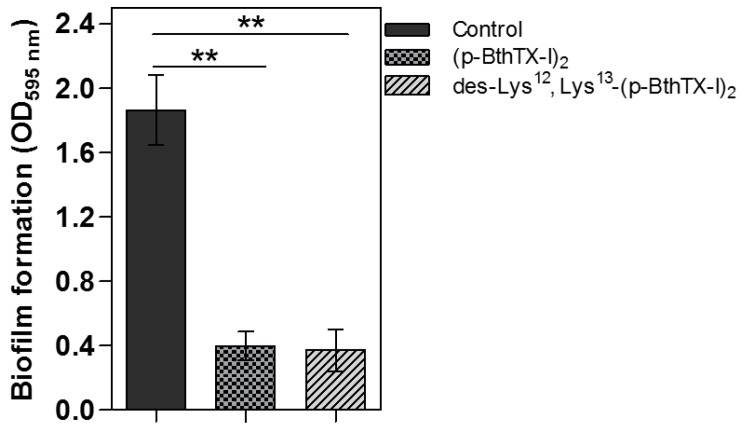
Biofilm eradication activities of (p-BthTX-I)_2_ and des-Lys^12^/Lys^13^-(p-BthTX-I)_2_. *S. epidermidis* ATCC 35984 growth observed after treatment with the peptides at 512 µM (** *p* < 0.01). The columns represent mean ± standard deviation (SD) of 16 replicates for each treatment.

Next, the capacity of the peptides to eradicate biofilms formed by *S. epidermidis* was evaluated. The result showed that both peptides were able to degrade the biofilms formed by *S. epidermidis* ATCC35984 with similar effectiveness (78.6% and 80% for (p-BthTX-I)_2_ and des-Lys^12^/Lys^13^-(p-BthTX-I)_2_, respectively; Figure 4).

**Figure 5:**
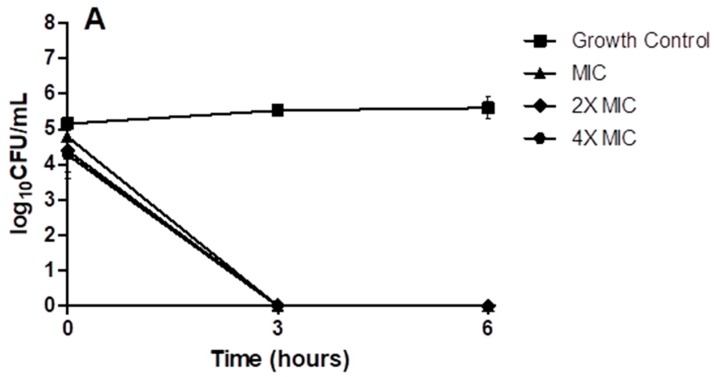

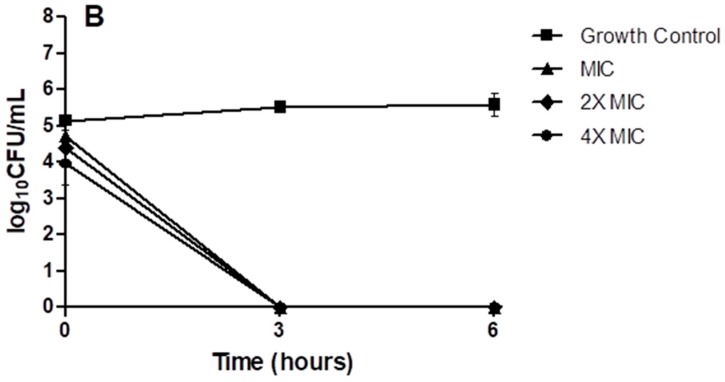
Time-kill curves of *S. epidermidis* ATCC 35984 incubated in the presence of (p-BthTX-I)_2_ and des-Lys^12^/Lys^13^-(p-BthTX-I)_2_. The bacteria were cultured in cation-adjusted BBL Mueller–Hinton II broth (CAMHB) containing 1×, 2×, or 4× MIC concentrations of either (**A**) (p-BthTX-I)_2_ or (**B**) des-Lys^12^/Lys^13^-(p-BthTX-I)_2_ as indicated. All data points represent the mean of two independent experiments. CFU: Colony Forming Units.

To confirm the bactericidal activity of (p-BthTX-I)_2_ and des-Lys^12^/Lys^13^-(p-BthTX-I)_2_ against the planktonic form of *S. epidermidis* ATCC35984, time-kill assays were performed. The results showed that at all concentrations tested (1×, 2×, and 4× minimum inhibitory concentration, MIC) of (p-BthTX-I)_2_ (Figure 5A) and des-Lys^12^, Lys^13^-(p-BthTX-I)_2_ (Figure 5B) were capable of eradicating the bacteria in the initial minutes of incubation, as evidenced by the log_10_ Colony Forming Units (CFU) /mL value differing from that of the growth control at time 0.

## 3. Discussion

In a previous study, Santos-Filho et al. synthesized and characterized the peptide p-BthTX-I (KKYRYHLKPFCKK) and its disulfide-linked dimeric form (p-BthTX-I)_2_ [19]. These peptides exhibited antimicrobial activity against *E. coli* ATCC25922 (MIC values of 16 and 4 µM for p-BthTX-I and (p-BthTX-I)_2_, respectively) and *S. aureus* ATCC25923 (MIC values of 64 and 32 µM for p-BthTX-I and (p-BthTX-I)_2_, respectively) and were nontoxic to *C. albicans* ATCC18804, erythrocytes, epithelial cells, and macrophages, indicating a potential specificity against prokaryotic cells. Furthermore, replacement of cysteine to a serine in the peptide attenuated its antibacterial activity. Thus, it was concluded that dimerization was crucial for the antimicrobial activity of p-BthTX-I and that the result of the monomeric form was due to the fast dimerization of the peptide in the culture medium [19]. The effects of dimerization on the antimicrobial peptide remain unclear, although previous studies have indicated increases or decreases in antimicrobial activity [7,20,21,22,23].

Concerns have been raised regarding antimicrobial peptide administration into the circulatory system; in vitro assays have suggested that the peptides are usually less effective, owing to a lack of resistance against serum proteases, which causes proteolytic degradation and low activity under the physiological conditions [24,25]. Because of these limitations, development of antimicrobial peptides for clinical usage has been restricted [11]. The development of different drug delivery systems could aid in administering antimicrobial peptides, enhancing their half-lives, maintaining and improving activities against specific targets, and decreasing toxicity.

Dimeric peptides are used as an alternative approach for enhancing half-life and increasing biological activity because they are less susceptible to protease degradation than monomeric peptides [19,22,26]. Thus, serum stability assays were performed to analyze proteolysis of p-BthTX-I and (p-BthTX-I)_2_ in human blood. Analytical HPLC analyses indicated that after incubation with human serum for 30 min, both peptides were completely degraded. In addition, the stable product des-Lys^12^/Lys^13^-(p-BthTX-I)_2_ was characterized.

Although antimicrobial peptides could be used systemically, susceptibility to proteolytic degradation by proteases is a significant hindrance to the development of peptide-based therapy [24,27]. Serum exhibits extensive protease and peptidase activity [28], and peptidomic studies have shown diverse peptidases in human blood [29]. There are three major classes of peptidases in blood: (1) amino- and (2) carboxyexopeptidases, which cleave at the amino and carboxyl termini, respectively, and (3) endopeptidases, which cleave internal peptide bonds [30]. Based on this classification and the results presented herein, (p-BthTX-I)_2_ is likely degraded by blood carboxypeptidase B, which specifically hydrolyzes C-terminal lysine and arginine residues (lysine carboxy-exopeptidases) [31].

The antimicrobial activity of the synthesized peptides, including des-Lys^12^/Lys^13^-(p-BthTX-I)_2_, the primary degradation product, was evaluated (Table 2). Similar to (p-BthTX-I)_2_ [19], all of the peptides exhibited antibacterial activity. Furthermore, they do not exhibited antifungal activity against *C. albicans* or toxicity against erythrocytes, suggesting that these peptides have promising therapeutic potential owing to their specificity for prokaryotic cells.

Additionally, circular dichroism (CD) spectroscopy was performed for all the peptides to evaluate the association between their antibacterial activity and structure. The peptides had similar spectra and had predominantly random coil content (data not shown). Our previous work showed that the parent peptide (p-BthTX-I)_2_ does not interact with membranes [19] (a common trait for peptides with high random coil content). Our data indicated that the peptide could not act on membrane mimetics as their mechanism of action (data not shown); this has been discussed previously [19]. Therefore, a detailed assessment of this peptide could promote a new class of antimicrobial compounds.

There are several concerns regarding the serum stability of (p-BthTX-I)_2_. After administration into the circulatory system, the antimicrobial effects of the peptide against bacteria may be faster than its degradation rate. Moreover, despite degradation of the dimeric peptide, the generated product also exhibited antibacterial activity.

P-BthTX-I, (p-BthTX-I)_2_, and des-Lys^12^/Lys^13^-(p-BthTX-I)_2_ exhibited antimicrobial activity against a variety of bacteria, including multidrug-resistant clinical strains (Table 3). Importantly, antimicrobial activity against resistant bacteria was analyzed using cation-adjusted MH broth. Development of antimicrobial peptides has been hindered by several problems, such as salt sensitivity, as antimicrobial activity can be altered by salts (inactivation or increased MIC) [32,33,34,35,36]; thus, this step was an important consideration for testing the peptide activity. Of the bacterial strains tested, the panel of multidrug-resistant clinical strains was critical for determining the therapeutic potential of these peptides. *S. aureus* SA16, SA33, SA88, and SA90 are methicillin-resistant *S. aureus* (MRSA) strains isolated from infected Brazilian patients and belong to the widespread clones ST5/105SCCmecII [37]. SA33 is also resistant to tigecycline, whereas SA88 is also daptomycin heteroresistant. MRSA has become one of the most important nosocomial pathogens worldwide, capable of causing a variety of hospital infections [38]. Hospital infections have been steadily increasing over the last few decades and are now mainstream due to the emergence of drug-resistant bacterial strains [39]. Among the most prevalent bacteria causing nosocomial infections, *K. pneumoniae*, can lead to serious infections, including urinary tract infections, hospital-acquired pneumonia, intra-abdominal infections, wound infections, and primary bacteremia [40]. (p-BthTX-I)_2_ was also active against *K. pneumoniae* ATCC700603, ATCCBAA1705, and NDM-1 strains, with the last two capable of producing β-lactamases [41]. *K. pneumoniae* ATCCBAA1705 produces *K. pneumoniae* carbapenemase (KPC), whereas NDM-1 produces a New Delhi Metallo-β-lactamase 1 (NDM-1). Moreover, *K. pneumoniae* ATCC700603 produces SHV-18, a β-lactamase that renders this strain resistant to several β-lactam antibiotics.

The peptides also were active against *E. faecium* and *S. epidermidis*. Normally, *E. faecium* has no adverse effects on the host, however, it can cause endocarditis, bacteremia, urinary tract infections, and meningitis, primarily in immunocompromised patients [42]. *Enterococcus* spp. are particularly relevant to the medical community owing to their incidence of antibiotic resistance [43]. *E. faecium* VRE16 is a high-risk, vancomycin-resistant enterococci clone of ST412 isolated from nosocomial infected patients worldwide [44], whereas the HBSJRP8 strain is daptomycin resistant.

*S. epidermidis* is primarily known as an innocuous, commensal bacterium on human skin. However, antibiotic-resistant *S. epidermidis*, mainly methicillin-resistant (MRSE), has gained increasing medical relevance [45,46]. Moreover, it has emerged as a major nosocomial pathogen associated with infections of implanted medical devices because of its biofilm formation properties [47]. Staphylococcal biofilms are characterized as a multicellular aggregate surrounded by a self-produced extracellular matrix consisting of proteins, polysaccharides, and extracellular DNA (eDNA; originating from the bacteria autolysis) [48,49]. The biofilm-forming cells can adhere to a variety of surfaces and are the major cause of nosocomial infections that colonize biomedical devices, such as respirators, catheters, prosthetic heart valves, and orthopedic devices [50,51]. Biofilms are a defense mechanism within bacteria, rendering them up to 1000 times more resistant to antimicrobial agents than their planktonic counterparts [52]. High levels of resistance can be due to delayed penetration of antimicrobial agents and changes in the metabolic rates of the microorganisms [53].

Biofilm eradication activity was analyzed against *S. epidermidis* ATCC35984, which is known to produce strong biofilms, isolated from a patient with intravascular catheter-associated sepsis [54,55] (Figure 4). Our data indicated that both (p-BthTX-I)_2_ and des-Lys^12^/Lys^13^-(p-BthTX-I)_2_ were effective against this strain (MIC of 16 and 32 µmol/L, respectively), suggesting that both molecules have therapeutic potential for developing new drugs.

## 4. Materials and Methods

### 4.1. Peptide Synthesis

Solid-phase peptide synthesis (SPPS) was performed following an Fmoc (9-fluorenylmethyloxycarbonyl)-based protocol using Fmoc-Lys(Boc)-Wang resin [56]. Coupling and deprotection were evaluated with ninhydrin [57]. To obtain the dimeric peptides, air oxidation was performed according to the previously described method [19].

### 4.2. Electrospray Mass Spectrometry

To confirm the identity of the peptides, diluted samples were analyzed by direct infusion mass spectrometry using an amaZon ion trap mass spectrometer (Bruker Daltonics, Billerica, MA, USA) coupled to a syringe pump (2.5–10 µL/min) in a positive electrospray ionization mode (ESI).

### 4.3. Reverse Phase Chromatography

Crude peptides were purified by semi-preparative HPLC on a Shimadzu system (Tokyo, Japan) using a C18 reversed phase column (10 × 250 mm, Phenomenex, Torrance, CA, USA). Solvent A contained 0.045% trifluoroacetic acid (TFA) in water. Elution was achieved using a linear gradient (5–35%) of solvent B (0.036% TFA in acetonitrile) for 120 min at 5 mL/min. The purity of the peptides was determined by analytical HPLC on a Shimadzu system using a C18 column (4.6 × 150 mm, Phenomenex, Torrance, CA, USA) with a linear gradient of solvent B (5–95%) for 30 min at 1 mL/min. Liquid chromatography-mass spectrometry (LC/MS) was performed using a C18 column (2.0 × 30 mm, Shimadzu) attached to analytical HPLC and an amaZon ion trap mass spectrometer (Bruker Daltonics) using ESI. Peptides were eluted using a linear gradient of solvent B (5–95%) for 15 min at 0.2 mL/min.

### 4.4. Serum Stability Assay

Peptide stability assays were performed in diluted serum as previously described [24,25] with minor modifications. First, 2 mL of 25% human male serum was centrifuged at 3000× *g* for 10 min; the supernatant was collected and incubated for 15 min at 37 °C. Next, peptides were added to the serum to a final concentration of 100 µM. Then, 200 µL of samples were collected at different time points during the assays, which were performed in duplicate. Analyses were performed by LC/MS and analytical HPLC, as described above.

### 4.5. Circular Dichroism Spectroscopy

CD spectra of peptides were recorded from 190 to 250 nm at room temperature with a J-815CD spectrophotometer (Jasco Co., Tokyo, Japan) under continuous nitrogen flush using 1-mm path length quartz cuvettes. To compare the secondary structures of the peptides, spectra were obtained in an aqueous solution (phosphate buffer saline, PBS), in a secondary structure-inducing solvent (trifluoroethanol, TFE), and in lysophosphatidylcholine (LPC). The peptide concentration was 60 µM. CD spectra were typically the average of six scans, measured in millidegrees.

### 4.6. Hemolysis Assay

Peptide hemolysis assays were performed as previously described by Castro et al. [58]. Briefly, freshly prepared human red blood cells (RBCs) were washed three times with 0.01 M Tris-HCl (pH 7.4) containing 0.15 M NaCl (Tris-saline). A suspension of 1% (*v*/*v*) erythrocytes was made with packed RBCs resuspended in Tris-saline. Peptides were dissolved in Tris-saline at an initial concentration of 128 mM and serially diluted in the same buffer to determine the concentration that triggered 50% hemolysis (HC_50_). As the positive control (100% lysis), 1% (*v*/*v*) Triton X-100 solution was used. After 1 h of incubation at 37 °C, the samples were centrifuged at 3000× *g* for 5 min. Then, 100 µL aliquots of the supernatant were transferred to 96-well microplates, and the absorbance was determined at 405 nm. The assay was performed in triplicates.

### 4.7. Fluorescence Spectroscopy

Fluorescence data acquisition was performed using an Eclipse spectrofluorometer (Varian Cary, Agilent Technologies, Santa Clara, CA, USA). The excitation was 276 nm to irradiate tyrosine groups, and the emission spectra were measured from 294 to 365 nm. Fluorescence spectra were obtained using peptide concentrations of 30 µM to a final volume of 600 µL. For studies of peptide-micelle interactions, titration of the peptides was performed in the zwitterionic detergent LPC. The peptide concentrations ranged from 0 to 10 mM in Tris-HCl, 0.01 M NaCl, pH 7.4. Fluorescence intensity and maximum emission wavelength data were obtained.

### 4.8. In Vitro Evaluation of the Antimicrobial Activity

The minimum inhibitory concentrations (MIC) were determined following the Clinical and Laboratory Standards Institute (CLSI) recommendations [59]. Antibacterial and antifungal activity tests were performed using the microdilution method. For initial screening, peptides were tested against *C. albicans* ATCC18804, *E. coli* ATCC25922, and *S. aureus* ATCC25923. Briefly, bacterial cells in MH broth (80 μL aliquots containing 1.5 × 10^7^ CFU) were incubated with synthetic peptides (serial dilution from 512 to 1 μM) dissolved in Milli-Q water. After incubation for 24 h at 37 °C, the microtiter plates were analyzed visually by addition of resazurin. For the antifungal assays, the culture medium was RPMI 1640 buffered with l-glutamine (pH 7.2), 0.165 M morpholinepropanesulfonic acid (MOPS), and 2% glucose. Cell suspensions (final concentration of 1 × 10^3^–2.5 × 10^3^ CFU/mL) were inoculated on a microdilution plate previously prepared with serially diluted synthetic peptides (128–1 μM). The plates were incubated for 48 h at 37 °C. Each assay was performed in triplicates. Additionally, amphotericin B and fluconazole were used as the control drugs. MIC was defined as the lowest concentration of peptide at which no growth was detected.

After confirming antibacterial activity, the peptides were tested against gram-positive and gram-negative multidrug-resistant bacteria, including strains of clinical origin. The peptides, p-BthTX-I, des-Lys^12^/(p-BthTX-I)_2_, and Lys^13^-(p-BthTX-I)_2_ were serially diluted (512–1 μM) in cation-adjusted BBL MH II broth (CAMHB; Becton, Dickson and Co., Sparks, MD, USA) as described by CLSI. Bacteria at 5.0 × 10^5^ CFU/mL were added to the media and incubated for 24 h at 37 °C. The microtiter plates were analyzed visually, and MIC was defined as the lowest concentration that completely inhibited bacterial growth. Assays were performed in duplicates, and sterile broth was used as the negative control. To determine the minimal bactericidal concentration (MBC), 100 µL from each well of the MIC assay plates that inhibited bacterial growth were sub-cultured onto MH agar (MHA) plates. The plates were incubated for 24 h at 37 °C, and MBC was defined as the lowest concentration of the peptides that resulted in 99.9% bacterial death. If the ratio of MBC/MIC was >4, the activity of the peptide was considered bacteriostatic, as described previously [60].

### 4.9. Biofilm Eradication

The biofilm eradication abilities of (p-BthTX-I)_2_ and des-Lys^12^/Lys^13^-(p-BthTX-I)_2_ were evaluated as previously described [61]. Briefly, *S. epidermidis* ATCC35984, known to produce biofilms, was cultured for 18 h on brain heart infusion broth (BHI; KASVI, Curitiba, PR, Brazil) supplemented with 0.75% glucose (*w*/*v*). Bacterial suspension was adjusted to an OD_600_ of 1 and then diluted to 1:40 in the same broth. Next, 200 µL of the bacterial dilution was added per well to a 96-well plate and incubated for 24 h at 37 °C. After bacterial adhesion, the plate was washed three times in PBS (pH 7.4), and the bacteria cells were incubated with either only the media (positive control) or with 512 µM peptides, des-Lys^12^/(p-BthTX-I)_2_, and Lys^13^-(p-BthTX-I)_2_, at 37 °C for 24 h. The samples were then washed in PBS before staining with crystal violet (0.2% *w*/*v*), and peptide activities were evaluated at 595 nm in a microplate reader (Polaris, Celer, Belo Horizonte, Minas Gerais, Brazil). A total of 16 experimental replicates were performed for each condition. One-way ANOVA was used to compare the absorbance values of the peptide treatments with the positive control. *p* < 0.05 was considered statistically significant.

### 4.10. Time-Kill Kinetics

The bactericidal activities of (p-BthTX-I)_2_ and des-Lys^12^/Lys^13^-(p-BthTX-I)_2_ against planktonic cells of *S. epidermidis* ATCC35984 were analyzed by a time-kill assay, as described previously [62]. Briefly, a bacterial suspension cultured for 18 h in BHI broth was adjusted with sterile 0.9% NaCl to 1.0 McFarland turbidity standard (approximately 3.0 × 10^8^ CFU/mL). The standardized bacterial suspension was diluted to 1:5 in CAMHB to approximately 6.0 × 10^7^ CFU/mL. Next, 100 µL of the adjusted bacterial suspension was added to 10 mL of CAMHB containing 1×, 2×, and 4× MIC concentrations of des-Lys^12^/(p-BthTX-I)_2_ or Lys^13^-(p-BthTX-I)_2_, and the final concentration was approximately 6.0 × 10^5^ CFU/mL. Immediately after inoculation, tubes were vortexed, and 100 µL was eluted from each tube and serially diluted in sterile 0.9% NaCl. Dilutions were plated on BHI agar (six replicates of 15 µL each) and incubated for 24 h at 37 °C for the zero time point. Tubes were incubated at 37 °C under constant agitation, and 100 µL aliquots were eluted after 3 and 6 h. Serial dilutions were plated on BHI agar and incubated for 24 h at 37 °C. Colonies were counted, and the results were recorded as log_10_ CFU/mL. A decrease in value of ≥3 log_10_ CFU/mL was considered bactericidal. Bacteria cultured in the absence of the peptides were used as the positive control and sterile broth was used as the negative control. The experiment was performed twice (total of two biological replicates).

## 5. Conclusions

In this study, the serum stability of the peptide (p-BthTX-I)_2_ was investigated. Encouragingly, the stable degradation product des-Lys^12^/Lys^13^-(p-BthTX-I)_2_ exhibited activity similar to that of the dimeric form (p-BthTX-I)_2_. These data suggest that after proteolysis of the parent peptide, the product retains antibacterial activity. In particular, des-Lys^12^/Lys^13^-(p-BthTX-I)_2_ is a potential peptide for antibacterial drug design because shorter peptides are cheaper to produce synthetically. Therefore, these peptides can be used as models for new drugs to target multidrug-resistant infections worldwide.

## Figures and Tables

**Table 1:** Characteristics of the synthetic peptides.

Peptide	Sequence													RT (min) ^a^	Mass
(p-BthTX-I)_2_	K	K	Y	R	Y	H	L	K	P	F	C	K	K	9.18	3476.2
des-Lys^13^-(p-BthTX-I)_2_	K	K	Y	R	Y	H	L	K	P	F	C	K	-	10.91	3219.9
des-Lys^12^, Lys^13^-(p-BthTX-I)_2_	K	K	Y	R	Y	H	L	K	P	F	C	-	-	11.35	2963.6
des-Lys^1^, Lys^2^-(p-BthTX-I)_2_	-	-	Y	R	Y	H	L	K	P	F	C	K	K	10.87	3219.9
des-Lys^1^-(p-BthTX-I)_2_	-	K	Y	R	Y	H	L	K	P	F	C	K	K	10.69	2963.6

^a^ Values obtained from analytical HPLC with C18 reverse phase column: 5–95% of solvent B for 30 min. Solvent A: 0.045% TFA in water. Solvent B: 0.036% TFA in acetonitrile. The peptides were eluted at a flow rate of 1.0 mL/min and detected at 220 nm. RT: retention time.

**Table 2:** Biological activities of the synthetic peptides.

Strain	MIC (µM)				
	(p-BthTX-I)_2_	des-Lys^13^-(p-BthTX-I)_2_	des-Lys^12^, Lys^13^-(p-BthTX-I)_2_	des-Lys^1^, Lys^2^-(p-BthTX-I)_2_	des-Lys^1^-(p-BthTX-I)_2_
*E. coli*	4	32	16	64	16
*S. aureus*	64	128	128	64	128
*C. albicans*	>128	>128	>128	>128	>128
HC_50_	>128	>128	>128	>128	>128

MIC: Minimum inhibitory concentration. HC_50_: The average value of the peptide concentration that produced 50% hemolysis.

**Table 3:** Biological activities of the synthetic peptides (p-BthTX-I)_2_ and des-Lys^12^/Lys^13^-(p-BthTX-I)_2_.

Bacterial Strains	p-BthTX-I		(p-BthTX-I)_2_		des-Lys^12^/Lys^13^-(p-BthTX-I)_2_	
	MIC (µM)	MBC (µM)	MIC (µM)	MBC (µM)	MIC (µM)	MBC (µM)
*S. epidermidis* ATCC35984	128	512	16	64	32	32
*S. aureus* ATCC25923	>512	N.D.	512	512	128	256
*S. aureus* SA16	512	512	>512/256′	512	128	128
*S. aureus* SA33	512/>512′	>512	256/512′	512	128	256
*S. aureus* SA88	512	>512	256	256	256	512
*S. aureus* SA90	512/>512′	512	128	256	128/512′	512/512′
*E. faecium* VRE16	128	>512	32	>512	16	64
*E. faecium* HSJRP8	32	>512	8	>512	8	32
*K. pneumoniae* ATCC700603	>512	N.D.	64	64	128/64	256/128
*K. pneumoniae* ATCCBAA1705	>512	N.D.	256	512	32	64
*K. pneumoniae* NDM-1	>512/512′	>512	256	512	256	512
*E. coli* ATCC35218	512	>512	512	512	64	256
*E. coli* CA4	>512	N.D.	256	512	64	128

MIC: Minimum inhibitory concentration. MBC: Minimal bactericidal concentration. N.D.: not determined.
